# A University-Led Take-Back Program for Pharmaceutical Waste Management: Eleven Years of Real-World Evidence on Medication Non-Use and Disposal Patterns

**DOI:** 10.3390/pharmacy14020042

**Published:** 2026-03-03

**Authors:** Alejandra E. Hernández-Rangel, Gustavo A. Hernández-Fuentes, Iván Delgado-Enciso, Hortensia Parra-Delgado, Jesús E. Castrejón-Antonio, Héctor R. Galván-Salazar, Alicia Olvera-Montejano, José Guzmán-Esquivel, Fabian Rojas-Larios, Josuel Delgado-Enciso, César G. Silva-Vázquez, Uriel Díaz-Llerenas, Juan M. Sánchez-Galindo, Mario A. Alcalá-Pérez, Daniel A. Montes-Galindo

**Affiliations:** 1 Department of Molecular Medicine, School of Medicine, University of Colima, Colima 28040, Mexico; alejjandra49@gmail.com (A.E.H.-R.); ghfuentes@ucol.mx (G.A.H.-F.); ivadelga@fiu.edu (I.D.-E.); frojas@ucol.mx (F.R.-L.); cesar.silva2359@alumnos.udg.mx (C.G.S.-V.); juanmanuelsg02@gmail.com (J.M.S.-G.); 2 Faculty of Chemical Sciences, University of Colima, Coquimatlan 28400, Mexico; hparra@ucol.mx; 3 State Cancerology Institute of Colima, Health Services of the Mexican Social Security Institute for Welfare (IMSS-BIENESTAR), Colima 28085, Mexico; hector_rgs@hotmail.com; 4 Robert Stempel College of Public Health and Social Work, Florida International University, Miami, FL 33199, USA; 5 Facultad de Ciencias Biológicas y Agropecuarias (FCBA), Autopista Colima-Manzanillo Km. 40, La Estación, Tecomán 28930, Mexico; jcastrejon3@ucol.mx; 6 Tecnológico Nacional de México, Campus Colima, Villa de Álvarez 28976, Mexico; alicia.olvera@colima.tecnm.mx; 7 Clinical Epidemiology Research Unit, Mexican Institute of Social Security, Villa de Alvarez, Colima 28984, Mexico; jose.esquivel@imss.gob.mx; 8 Foundation for Ethics, Education, and Cancer Research of the State Cancer Institute of Colima AC, Colima 28085, Mexico; josuel7@hotmail.com; 9 Molecular Medicine Laboratory, Academic Unit of Human Medicine and Health Sciences, Autonomous University of Zacatecas, Zacatecas 98160, Mexico; urieldiazllerenas@gmail.com (U.D.-L.); marioalcalaperez@uaz.edu.mx (M.A.A.-P.); 10 Carrera de Medicina Integral y Salud Comunitaria, Universidad para el Bienestar Benito Juárez García (UBBJ)-Sede Armería, Degollado 428, Ejido Armería, Armería 28305, Mexico

**Keywords:** pharmaceutical waste, medication take-back programs, therapeutic adherence, real-world evidence, ATC classification, self-medication, chronic disease medicines, environmental health

## Abstract

Background/Objectives: Improper medication use, premature treatment discontinuation, and inadequate disposal contribute to irrational drug consumption and environmental contamination. Although pharmaceutical take-back programs have expanded globally, real-world evidence on household medication accumulation in academic and community settings remains limited. This study aimed to describe longitudinal patterns of medication collection during an eleven-year university-based take-back campaign, with detailed pharmacological characterization available for selected post-pandemic years. Methods: Real-world data were analyzed from a sustainable medication take-back campaign conducted annually at the University of Colima between 2015 and 2025. Expired or unused medications were voluntarily returned by students and community members. Total collected weight was recorded for all years, while detailed classification by dosage form, Anatomical Therapeutic Chemical (ATC) group, and Mexican regulatory fraction (Fractions II, IV, V, and VI) was performed for years with complete records (2023–2025). All materials were disposed of through an authorized hazardous-waste company in compliance with NOM-052-SEMARNAT-2005. Descriptive analyses were performed using SPSS version 29.0. Results: Approximately 3.9 tons of pharmaceutical products were collected over eleven years, reflecting persistent household accumulation of unused or expired medicines. In the years with detailed analysis, oral solid dosage forms predominated. In 2025, ATC groups M, A, and C were most frequently returned, consistent with medications used for chronic conditions. Therapeutic composition varied annually, with NSAIDs/analgesics predominating in 2023–2024 and antibiotics in 2025. Across analyzed years, 5–7% of collected items corresponded to non-medication products. Conclusions: This long-term campaign provides valuable real-world evidence on medication non-use and disposal, highlighting ongoing challenges in rational medicine use, treatment continuity, and environmentally responsible pharmaceutical waste management.

## 1. Introduction

The improper use, inappropriate disposal, and premature abandonment of medications represent growing challenges for healthcare systems and the environment. The accumulation of expired or unused pharmaceuticals in households has been widely reported as a relevant source of pharmaceutical waste and a preventable contributor to environmental contamination and unintentional human exposure [1,2,3]. In addition to its environmental implications, the presence of unused medicines in domestic settings may reflect inefficiencies in prescribing, dispensing, storage, or treatment continuation, although for OTC products, household availability can support safe self-care and reduce pressure on the healthcare system [4,5,6,7].

Although unused or returned medications are not a direct measure of therapeutic adherence, their accumulation and subsequent disposal provide indirect real-world information on patterns of non-use, treatment discontinuation, and medication abandonment at the community level [2,3,7]. Previous studies have associated pharmaceutical waste with factors such as therapeutic duplication, self-medication, and communication gaps between patients and healthcare professionals; however, empirical evidence derived from long-term, community-based datasets remains limited, particularly in low- and middle-income settings [4,5,6,7]. Moreover, most available studies focus either on environmental contamination or on medication-use behavior, rarely integrating both dimensions using real-world data.

Within this context, universities are uniquely positioned to implement socially relevant initiatives that simultaneously promote responsible practices and generate empirical data on medication disposal patterns [8,9,10,11]. University-led take-back campaigns, frequently supported by student participation and outreach activities, have been described as effective mechanisms for facilitating the collection of expired or unused medicines while contributing to professional training in pharmaceutical regulation, hazardous waste management, and public health awareness [12,13,14,15]. In Mexico, expired medications are classified as hazardous waste under NOM-052-SEMARNAT-2005 and must be handled and disposed of under specific regulatory guidelines; nevertheless, inappropriate storage and disposal practices remain common among the general population [16].

Internationally, several countries have established consolidated medication return schemes, including pharmacy-based programs in Sweden and the United Kingdom, as well as national initiatives in Australia and Canada. These programs have demonstrated improvements in pharmaceutical waste management and increased public awareness regarding appropriate medicine disposal [17,18,19,20]. Parallel environmental evidence underscores the relevance of such interventions: global monitoring studies have detected pharmaceutical residues in the vast majority of surveyed rivers, with concentrations potentially harmful to aquatic organisms in a substantial proportion of sites [21,22,23]. Although household disposal may contribute a relatively small fraction of total pharmaceutical loads in aquatic environments for some compounds, estimated at approximately 1–3% in Nordic countries, it remains significant due to its preventable nature and the persistence and toxicity of certain active ingredients [24,25,26,27]. Consequently, community-based collection campaigns are increasingly recognized as complementary strategies to reduce avoidable environmental emissions while generating data that can inform prevention and education efforts [28].

In contrast to these well-established national schemes with >80% territorial coverage (e.g., Sweden’s Apotekets insamling), safe disposal strategies in Latin America and Mexico are still in the early stages of implementation, with limited infrastructure and low coverage of collection containers per 100,000 inhabitants, resulting in low overall collection rates compared with Europe [29,30]. Household storage of leftover and non-prescribed medicines is common in the region, and improper disposal (e.g., in household garbage or wastewater) remains widespread [31,32]. Furthermore, high prevalence of self-medication and over-the-counter use in Mexico—reported at well over half of the population in some studies—amplifies the volume of unused or expired medicines retained in households and underscores the need for systematic take-back initiatives [29,30]. Community-based collection campaigns are increasingly recognized as complementary strategies to reduce avoidable environmental emissions while generating data that can inform prevention, education, and rational use efforts in low- and middle-income country contexts with high self-medication practices [33,34].

The primary operational objective of the campaign was to facilitate the safe collection and disposal of expired or unused medications at designated collection points. Over time, this initiative generated a longitudinal dataset spanning 2015 to 2025 that documents annual quantities of pharmaceutical waste collected, reflecting real-world patterns of medication accumulation and disposal. Detailed pharmaceutical characterization—by dosage form, regulatory classification, and therapeutic group—was systematically available for the most recent three years of the program.

Returned medications intake-back programs do not constitute a direct measure of therapeutic adherence. Instead, they should be interpreted as proxy indicators of medication non-use, premature discontinuation, or household accumulation. The presence of unused or expired products reflects failures at different stages of the medication-use process—such as non-initiation, early discontinuation, therapeutic duplication, or changes in prescribed regimens—rather than confirmed non-adherence at the individual patient level. Accordingly, the findings of this study are discussed as indirect signals of challenges in medication use and continuity of treatment, rather than as clinical adherence outcomes.

Therefore, the aim of this study is to present a longitudinal analysis of real-world data derived from a sustainable, university-led medication take-back campaign, describing temporal trends in the volume of pharmaceutical waste collected and the composition of returned products. By characterizing patterns of non-use and disposal, this work seeks to contribute evidence relevant to pharmaceutical waste management and to provide indirect insights into medication use and treatment discontinuation at the community level, while highlighting opportunities for future educational and public health interventions.

## 2. Materials and Methods

### 2.1. Campaign Design and Dissemination Strategy

The medication take-back campaign was organized and implemented by faculty members and eighth-semester students from the Faculty of Chemical Sciences at the University of Colima as part of an academic outreach activity. The primary operational objective of the campaign was to facilitate the safe collection of expired or unused medications and to prevent their improper disposal, thereby reducing potential environmental contamination and risks associated with inappropriate use.

The campaign was conducted in the metropolitan area of Colima–Villa de Álvarez and nearby municipalities. Collection activities were carried out on a continuous basis throughout the year, supported by periodic dissemination actions, including informational flyers, brief community-oriented talks, and the placement of clearly identified collection containers at strategic locations.

Dissemination activities aimed to promote safe medication disposal and encourage the public to deposit unused or expired medicines in designated collection containers. One container was placed on each participating campus, in public health sector hospitals, and in selected public pharmacies. Containers complied with NOM-052-SEMARNAT-2005 for hazardous waste and were monitored by campaign organizers, following COFEPRIS recommendations for secure medicine collection [16]. These activities were not designed, validated, or evaluated as a formal educational intervention. No pre–post assessments, comparison groups, or structured evaluations of knowledge or behavior change were conducted. Accordingly, the study did not assess the impact of informational activities on participant behavior; the campaign was used solely as a source of observational data on returned pharmaceutical products.

Formal collaboration agreements were established with public healthcare institutions—including IMSS facilities and a Community Hospital—as well as multiple campuses of the University of Colima. These agreements ensured continuous access to collection containers both on university campuses and at participating healthcare facilities, allowing the campaign to operate actively and uninterrupted from 2015 to 2025.

### 2.2. Collection Timeline and Eligibility for Analysis

All medications brought to the collection points were accepted regardless of expiration status, including expired products, unused medicines still within their validity period, incomplete treatments, and discontinued therapies. For analytical purposes, only products meeting predefined identification and integrity criteria were included in the study database (see Section 2.3). These inclusion criteria were applied exclusively to classification and quantification and did not restrict collection or disposal. The source institution or location of the medications was recorded, but the identity of individual participants remained anonymous.

### 2.3. Inclusion and Exclusion Criteria

The inclusion and exclusion criteria described below were applied only for analytical classification and quantification of the collected medications. Medications were included in the analytical dataset if they met all of the following criteria: (1) presence of complete secondary packaging; (2) legible identification of medication name, active ingredient, batch number, and expiration date; and (3) adequate physical condition allowing correct pharmaceutical classification [16,29].

Products were excluded from analytical classification only if they consisted of: (1) loose blisters without secondary packaging; (2) unidentified or incomplete solid dosage forms; or (3) products that did not meet the regulatory definition of a medication under Mexican pharmaceutical standards.

Importantly, all collected items were retained and handled as part of the campaign, even if they did not meet analytical criteria. Products not meeting the classification criteria were not quantified or categorized but were transferred for final disposal through an authorized hazardous waste management company, in accordance with NOM-052-SEMARNAT-2005 [16,35,36].

### 2.4. Medication Classification

All collected medications were initially transported to the Coquimatlán campus, where they were consolidated, weighed, and recorded. Handling, transport, and classification were performed exclusively by trained personnel, including students and supervisors instructed in pharmaceutical safety, hazardous waste management, and relevant Mexican regulations. Personal protective equipment was used throughout all procedures, and medications were managed strictly as hazardous waste.

Solid oral dosage forms were received both as complete commercial packages and as partially used blister packs or loose individual units. Products were recorded as delivered, without standardization by the number of units per package. Quantification was based exclusively on total collected weight rather than individual unit counts, ensuring comparability across heterogeneous and incomplete returns.

Prior to weighing, external secondary packaging (e.g., cardboard boxes and paper inserts) was removed when present. However, primary containers (e.g., blisters, plastic bottles, glass vials, tubes) were retained and weighed together with the pharmaceutical contents, as these materials are disposed of jointly as hazardous pharmaceutical waste under Mexican regulations. Therefore, total mass reflects the pharmaceutical product along with its immediate containment.

At the analysis center, each product was evaluated against the predefined inclusion criteria (see Section 2.3). Products meeting the criteria were classified according to: (1) the Anatomical Therapeutic Chemical (ATC) classification system [37,38]; (2) pharmaceutical dosage form (e.g., tablets, capsules, oral solutions, injectables, powders, ointments) [39]; and (3) the applicable sanitary regulatory fraction under Mexican legislation (Fractions II, IV, V, and VI), with an additional “Not applicable” category for non-medication products [16].

Products not meeting analytical criteria were not classified or quantified but were immediately transferred for final disposal through an authorized hazardous waste management company, in accordance with NOM-052-SEMARNAT-2005 [16].

Under current Mexican regulations, Fraction III does not exist, as this category was eliminated in previous regulatory updates; therefore, the sequence proceeds directly from Fraction II to Fraction IV [16].

All classified data were entered into a centralized database designed specifically for this project to ensure standardized data management and reproducibility.

### 2.5. Data Availability Across the Study Period

The campaign remained operational from 2015 to 2025, and annual records of the total weight of collected pharmaceutical waste were available for the entire period. Detailed pharmaceutical classification—including dosage form, ATC code, and regulatory fraction—was performed only for the final three years of the study (2023–2025).

### 2.6. Regulatory and Ethical Considerations

This project constituted an environmental and operational waste management initiative focused on the collection and safe disposal of expired or unused medications. No human participants were recruited, and no identifiable personal, clinical, or behavioral data were collected. Approval and oversight were provided through the coordinated effort between the Faculty of Chemical Sciences and the University of Colima via its academic outreach and volunteer programs, in collaboration with public healthcare institutions in the state of Colima [5].

All collected materials were managed as hazardous waste in compliance with NOM-052-SEMARNAT-2005 and transferred to an authorized destruction facility, ensuring traceability, safety, and environmental protection [16].

### 2.7. Final Disposal of Collected Medications

After classification and data recording, all collected medications—including those excluded from analytical classification—were sent to an authorized hazardous waste destruction company with which the Faculty of Chemical Sciences maintains an active agreement. This procedure prevented landfill disposal or unauthorized handling and ensured environmentally responsible final destruction [40].

### 2.8. Statistical Analysis

Data analysis was descriptive and exploratory in nature. Annual totals were summarized as absolute weights (kg) and relative proportions (%), stratified by dosage form, Anatomical Therapeutic Chemical (ATC) group, and Mexican regulatory fraction. Percentages were calculated based on total collected mass rather than unit counts, as the primary objective of the campaign was environmental pharmaceutical waste characterization, and mass provided a standardized and operationally feasible metric across heterogeneous and partially used dosage forms. No inferential or hypothesis-testing statistics were performed, as the data derives from a voluntary, non-probabilistic collection process and are intended to characterize observed patterns rather than establish causal or statistically significant differences between years. Data management and descriptive summaries were conducted using IBM SPSS Statistics version 29.0 (IBM Corp., Armonk, NY, USA) [41].

## 3. Results

### 3.1. Collection Campaign and Promotion Strategy

The medication collection campaign has been implemented as a permanent, year-round initiative since 2015 as part of an educational strategy directed toward students in the Pharmaceutical and Biological Chemistry programs. Collection modules remain continuously available throughout the year, while promotional activities are conducted periodically within the academic community, including informational talks, dissemination through institutional social media, installation of visible collection modules within faculty facilities, and the active participation of student volunteers.

This strategy has increased awareness regarding the risks associated with prolonged storage and improper disposal of medications, while generating valuable real-world data for analyzing patterns of medication accumulation, non-use, and abandonment among university students and their families.

### 3.2. Trends in Collected Medications (2015–2025)

**Figure 1:**
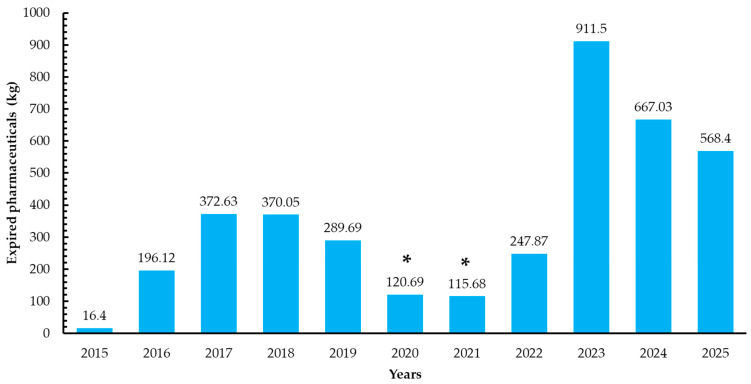
Bars represent the total kilograms of medications collected each year. Years marked with an asterisk (*) correspond to periods affected by mobility and interaction restrictions during the COVID-19 pandemic (2020–2021).

From 2015 to 2025, the campaign accumulated a total of 3876.06 kg of expired, unused, or discontinued medications, reflecting the magnitude of pharmaceutical waste generated at the community level (see Table 1). The annual amount collected varied considerably across the study period (Figure 1).

The initial years showed a progressive increase, reaching peaks in 2017 (372.63 kg) and 2018 (370.05 kg). Subsequently, a marked decrease was observed during the COVID-19 pandemic (2020–2021), coinciding with mobility restrictions and limited access to institutional facilities.

In 2023, the highest historical value was recorded (911.50 kg), likely associated with post-pandemic accumulation of stored medications and strengthened student participation strategies. In the most recent year analyzed (2025), the campaign collected 568.4 kg, representing the second-highest mass recorded since its implementation.

### 3.3. Distribution of Collected Pharmaceutical Dosage Forms (2023–2025)

All collected medications that met the analytical inclusion criteria were analyzed. Across the three years for which detailed pharmaceutical characterization was available (2023–2025), solid oral dosage forms clearly predominated among the collected medications (Table 1). Tablets consistently represented the largest proportion, accounting for 63.4% in 2023, 57.8% in 2024, and 59.6% in 2025. Capsules were the second most frequent solid form in 2023 and 2024, although their relative contribution declined markedly in 2025 (11.4%). Powders constituted a minor but stable fraction, ranging from 3.5% to 4.4% across the evaluated period.

Semisolid dosage forms (including ointments and creams) represented a small proportion of the total pharmaceutical waste and showed limited year-to-year variation, never exceeding 4.1% in any year.

Liquid dosage forms exhibited a clear decreasing trend over time. Solutions and injectable preparations declined from 8.2% of the total collected weight in 2023 to 3.5% in 2025, while suspensions remained relatively stable, contributing between 3.4% and 4.9%. Syrups followed a similar declining pattern.

In 2025, additional dosage forms not observed in previous years were documented, including ophthalmic solutions (2.6%) and transdermal patches (4.4%), reflecting an expansion in the diversity of collected products.

Finally, non-medication items accounted for a small fraction of the total collected weight, increasing from 0.4% in 2023 and 0.3% in 2024 to 4.4% in 2025. Overall, the analyzed dataset represents the complete set of medications that met the inclusion criteria, corresponding to approximately 99.6%, 99.7%, and 95.6% of the total collected weight in 2023, 2024, and 2025, respectively, while the remaining fraction, consisting of non-medication products, was transferred directly for final disposal without classification.

### 3.4. Regulatory Classification of Collected Medications (2023–2025)

In line with the predominance of solid oral dosage forms described in Section 3.3, the regulatory classification of the collected products revealed a clear dominance of Regulatory Fraction IV (over-the-counter medications) across all three evaluated years. This category accounted for 67.8% of the total collected weight in 2023, increased markedly to 82.35% in 2024, and declined to 68.4% in 2025 (Table 2).

Regulatory Fraction VI, corresponding to herbal medicines and dietary supplements, represented the second most frequent group, contributing between 15.69% and 20.9% of the total waste collected. The substantial presence of this fraction is consistent with the high proportion of non-prescription solid oral products identified in the dosage-form analysis.

Prescription-only medications (Fraction II) represented a small proportion of the total pharmaceutical waste in 2023 and 2024 (<1%) but showed a noticeable increase in 2025 (2.6%). Fraction V (homeopathic medications) remained relatively stable throughout the study period, ranging from 0.65% to 1.8%.

Fraction III was recorded only in 2024 (0.65%), reflecting transitional or limited regulatory classifications during that year.

Finally, the “Not applicable/non-medication” category accounted for 8.0% of the collected material in 2023 and 7.0% in 2025, while no such products were recorded in 2024. This category complements the dosage-form findings by highlighting the presence of non-pharmaceutical items stored and discarded together with medications.

### 3.5. Distribution by ATC Therapeutic Group (2023–2025)

Detailed pharmaceutical classification by ATC therapeutic group was performed for all collected medications that met analytical inclusion criteria during 2023–2025. Table 3 summarizes the distribution of collected pharmaceutical waste across major ATC groups and the “Without ATC code” category, which includes products that could not be reliably assigned to an ATC group due to incomplete labeling, deterioration, or insufficient identification at the time of classification.

Musculoskeletal system drugs (ATC group M) predominated in 2023 (38.1%), while other major groups—including alimentary tract and metabolism (A), cardiovascular system (C), and nervous system (N)—became more prominent in 2025, reflecting changes in the composition of collected medications over time. Non-medication products and items without ATC codes accounted for a variable proportion each year, highlighting the presence of improperly labeled or non-classifiable products.

Percentages are calculated relative to the total weight of pharmaceutical waste collected each year and are presented for descriptive purposes only. They do not always sum to 100% due to rounding and the presence of minor ATC groups not explicitly listed. Year-to-year differences should not be interpreted as statistical comparisons. All classifications were performed in accordance with current Mexican regulations for pharmaceutical waste management.

### 3.6. Distribution by Dosage Form and Regulatory Classification (2023–2025)

To integrate the dosage-form and regulatory analyses presented in Section 3.3 and Section 3.4, a cross-distribution assessment was conducted to explore the relationship between pharmaceutical form and regulatory status across the three evaluated years.

Solid oral dosage forms were primarily associated with non-prescription regulatory categories, particularly Fractions IV and VI, reflecting the predominance of over-the-counter medications and herbal or supplemental products within household accumulation patterns. Liquid and semisolid formulations showed a more heterogeneous regulatory distribution, including a limited contribution from prescription-only categories.

Although prescription medications represented a minor proportion overall, a modest increase was observed in 2025, coinciding with greater diversification of collected products. Non-medication items were identified across multiple dosage forms, highlighting persistent ambiguity in the differentiation between pharmaceutical and non-pharmaceutical health-related products.

This integrative approach complements the separate distributions previously described without duplicating the numerical data presented in Table 1 and Table 2.

When products were grouped by Mexican regulatory classification, non-prescription medications (Fraction IV) accounted for the largest proportion each year, confirming the central role of over-the-counter products in household accumulation and disposal. Herbal medicines and dietary supplements (Fraction VI) consistently represented a substantial share of the collected materials, ranging from approximately 16% to 21%, highlighting their frequent use and prolonged storage. Prescription-only medications (Fractions II and III) remained a minor component throughout the study period, although their relative contribution increased slightly in 2025. A small but stable fraction of collected items corresponded to products classified as non-medications under current regulations, such as nutraceuticals and similar products, indicating persistent ambiguity in public understanding of pharmaceutical versus non-pharmaceutical health products.

### 3.7. Categories with Greatest Absolute Change and Therapeutic Distribution of Collected Medications (2023–2025)

Analysis of absolute changes between consecutive years revealed that non-prescription medications (Fraction IV) exhibited the largest reduction in collected weight, decreasing by 160.5 kg between 2024 and 2025. Within the ATC classification, the most pronounced variation corresponded to the “No ATC code” category, which decreased markedly from 372 kg in 2024 to 30.1 kg in 2025, reflecting improved product identification and classification rather than a true change in therapeutic composition.

Regarding dosage forms, solutions and injectable solutions showed the greatest absolute decrease, declining by 78.1 kg from 2024 to 2025. These reductions contributed to the overall shift toward a higher relative proportion of solid oral forms in the final year.

The therapeutic composition of collected medications varied across the study period (Table 4). In 2023 and 2024, non-steroidal anti-inflammatory drugs (NSAIDs) and analgesics represented the largest share of returned products, whereas in 2025, antibiotics constituted the most prevalent category. Antiparasitic agents increased progressively over time, while antivirals showed a marked decline. Antifungal products exhibited moderate fluctuations, with a decrease in 2024 followed by a rebound in 2025. These changes indicate year-to-year variability in the types of medicines accumulated and discarded by households, rather than a consistent temporal trend, and underscore the heterogeneous nature of medication use and discontinuation patterns in the population studied.

## 4. Discussion

The present medicine collection campaign, conducted continuously over an eleven-year period (2015–2025), provides valuable insight into patterns of medicine storage, use, and disposal within an academic community and its surrounding households. Importantly, while annual data on the total weight of collected pharmaceutical waste were available for the entire campaign period, detailed pharmaceutical characterization (dosage form, regulatory fraction, ATC classification, and therapeutic category) was systematically performed only during the most recent three years (2023–2025). Consequently, interpretations related to pharmacological composition and regulatory or therapeutic patterns are restricted to this post-pandemic period.

The longitudinal analysis of total collected weight (Section 3.2) revealed marked interannual variability. The progressive increase observed between 2016 and 2018 likely reflects growing awareness and participation among students, whereas the decline during 2020–2021 coincides with mobility restrictions and reduced access to university facilities during the COVID-19 pandemic [42]. The pronounced peak observed in 2023 suggests a post-pandemic accumulation of unused medicines in households, combined with renewed outreach and student engagement. Similar trends have been reported in post–COVID-19 studies describing increased preventive medicine purchasing, treatment interruption, and stockpiling behaviors [42,43,44].

The detailed analysis of the 2023–2025 period showed a consistent predominance of solid oral dosage forms, particularly tablets and capsules (Section 3.3, Table 1), which are commonly associated with chronic disease management. This finding aligns with the regulatory classification results (Section 3.4, Table 2), over-the-counter medications (Fraction IV) represented the largest proportion across all three years. For these products, adherence is not the primary concern; rather, rational and appropriate use is the focus, particularly for painkillers and self-care medicines. Herbal products and dietary supplements (Fraction VI) were the second most frequent category. Adherence considerations remain primarily relevant for prescription-only therapies (Fraction II) used for chronic conditions. Together, these results suggest sustained patterns of self-medication, prolonged storage, and incomplete treatment courses.

Therapeutic classification according to the ATC system (Section 3.5, Table 3) further supports this interpretation. In 2025, the predominance of ATC groups M (musculoskeletal system), A (alimentary tract and metabolism), and C (cardiovascular system) reflects medicines commonly used for pain, metabolic disorders, and cardiovascular conditions—therapeutic areas in which adherence is critical to prevent complications. The return of these products indicates premature discontinuation, duplicated prescriptions, or lack of therapeutic follow-up, consistent with previously described adherence challenges [45,46]. Thus, while adherence issues are highlighted for prescription-only medicines, for OTC products, the focus should be on rational use and safe storage rather than strict adherence.

Complementary analysis of therapeutic categories (Section 3.7, Table 4) revealed year-to-year variability in the relative contribution of major drug classes. NSAIDs and analgesics predominated in 2023 and 2024, whereas antibiotics represented the largest proportion in 2025. These variations do not indicate statistically comparable trends but rather reflect changes in the composition of medicines accumulated and discarded each year. The observed decrease in antivirals and increase in antiparasitic and antifungal agents may be associated with post-pandemic shifts in prescribing practices and household medicine retention [47,48,49,50].

From a regulatory and safety perspective, the presence of prescription-only medicines (Fraction II) and herbal products or supplements (Fraction VI) in the collected waste (Section 3.7, Table 4) highlights potential risks related to inappropriate storage, misuse, and environmental contamination. Although these categories represent a smaller proportion of the total collected weight, their presence underscores the need for targeted educational strategies addressing medicines requiring stricter control and oversight [51,52,53,54]. In the Mexican context, herbal remedies and dietary supplements (Fraction VI) warrant attention because many are available over the counter, some lack proper identification, and a considerable number are used concomitantly with other medications. Therefore, these products can pose risks related to potential interactions, mismanagement, and regulatory ambiguity, highlighting the importance of educational interventions and careful disposal practices [55,56,57].

Additionally, approximately 5–7% of collected items were classified as “non-medications” under Mexican regulations [58]. This category includes nutraceuticals and herbal products perceived by users as therapeutic but legally classified as dietary supplements. Rather than reflecting classification errors, these finding underscores regulatory ambiguities and communication gaps between health authorities and the general population. These results point to the need for clearer public information regarding the legal status, therapeutic claims, and disposal requirements of such products [51,59].

From an environmental standpoint, the recovery of nearly 3.9 tons of pharmaceutical waste over eleven years represents a significant contribution to preventing soil and water contamination by active pharmaceutical ingredients. Beyond its environmental impact, this student-led initiative generates real-world evidence on medicine accumulation and disposal practices, reinforcing its value as both an educational and public health intervention [60,61].

Furthermore, the findings highlight the limited role of pharmacists in supervising medicine use in Mexico compared with healthcare models in Europe, Canada, and Australia, where community pharmacists actively contribute to adherence monitoring and rational medicine use [62,63]. Strengthening pharmacist participation in primary care and community-based interventions could substantially reduce pharmaceutical waste and improve therapeutic outcomes [64,65].

Several limitations should be acknowledged. Detailed pharmacological classification was available only for the post-pandemic period (2023–2025), limiting historical comparisons of medicine composition. The anonymous nature of the collection precludes identification of individual causes of treatment discontinuation or non-adherence, and the total weight of collected medicines does not directly reflect disease prevalence. Participation bias is also possible, as data derived only from individuals who voluntarily returned medicines [62,65,66]. In addition, this study was conducted at a single collection site within one country and represents a descriptive analysis without comparison across multiple geographic regions (semi-urban). Therefore, the findings may not be fully generalizable to other contexts with different demographic or regulatory conditions. However, the semi-urban setting provides relevant insight into medication accumulation patterns in transitional population environments. Finally, controlled substances were classified according to the applicable Mexican sanitary regulatory framework (Reglamento de Insumos para la Salud, 2021). Although no additional subclassification (e.g., specific narcotic or psychotropic categories) was performed, and expiration status or residual content within returned packages was not recorded, future medication take-back initiatives could incorporate more granular differentiation of controlled substances as well as detailed assessment of product validity and remaining contents. Such refinements would enhance cross-regulatory comparability and provide deeper insight into medication utilization patterns and waste generation dynamics beyond mass-based environmental characterization.

Despite these limitations, the continuity of the campaign offers a robust platform for future research. Integrating surveys, qualitative interviews, and digital adherence tools could enhance understanding of medicine disuse and support the development of targeted interventions aimed at reducing pharmaceutical waste and improving adherence.

### Policy Recommendations and Environmental Implications

Building on the findings of the present study, several strategies are recommended to reduce pharmaceutical waste and promote responsible medicine use at the community level. First, structured educational programs in schools and universities can increase awareness of proper medicine storage, adherence to prescribed therapies, and safe disposal practices. Early educational interventions are likely to instill long-term behavioral changes and improve household management of medicines.

Second, the establishment of permanent collection points in pharmacies would provide continuous, accessible avenues for the return of expired, unused, or discontinued medicines. International experience, such as the UK’s free pharmacy-based take-back programs, demonstrates that such initiatives can reduce household pharmaceutical waste by up to 20% [31,32]. Legislative support, including formal integration into the Mexican General Health Law, could standardize these programs nationally.

Third, fiscal or programmatic incentives may further encourage voluntary returns. For instance, tax deductions for medicines returned at authorized points or subsidies for licensed disposal companies can increase participation rates and ensure safe disposal. Scaling the Colima model to 50+ Mexican universities could potentially prevent the accumulation of ~50 tons of pharmaceutical waste annually, significantly reducing environmental exposure to active pharmaceutical ingredients (APIs). Based on estimated leaching rates of 5–20% into water bodies, the initiative may have avoided the release of ~195–780 kg of APIs into local rivers, highlighting tangible environmental benefits alongside educational and public health impacts [67,68,69].

In Mexico, medicines are classified into prescription-only and over-the-counter categories under the national sanitary regulatory framework and are primarily dispensed through community pharmacies. However, pharmacist oversight requirements differ from those in countries such as the United States and member states of the European Union, where continuous pharmacist supervision is mandatory. Clarifying this regulatory context is essential when considering the proposed role of community pharmacists as gatekeepers in promoting rational medicine use, antibiotic stewardship, and proper disposal practices.

Fourth, regulatory harmonization and enforcement should be considered within broader pharmaceutical stewardship strategies. Regulatory frameworks governing antibiotic dispensing vary internationally. In Mexico, systemic antibiotics legally require a medical prescription; however, prior to regulatory strengthening measures implemented in the past decade, over-the-counter access was more common due to inconsistent enforcement. In contrast, oral antibiotics have long required a prescription in the United States and across member states of the European Union, where dispensing without medical authorization is prohibited. Differences in regulatory oversight and enforcement may influence patterns of antibiotic accumulation, inappropriate storage, and subsequent disposal observed in medication take-back initiatives. Lastly, the rise in online medicine sales and consumer-driven purchasing may contribute to medication stockpiling and surplus, potentially affecting disposal patterns observed in take-back campaigns. Although this study did not assess purchasing channels, future research could explore this influence.

## 5. Conclusions

This eleven-year university-based medicine collection campaign demonstrates that such initiatives not only mitigate the environmental risks associated with improper pharmaceutical disposal but also provide valuable insight into patterns of medicine use, household accumulation, and treatment practices. While long-term trends in collected weight highlight sustained participation and post-pandemic effects, detailed analysis of the 2023–2025 period clarifies that over-the-counter medicines primarily reflect rational self-care practices, whereas adherence challenges are more relevant for prescription-only therapies used for chronic disease management. The findings emphasize the need to strengthen educational strategies, regulatory communication, and the role of pharmacists in community and academic settings. Overall, integrating medicine collection campaigns into health sciences training represents a sustainable and replicable approach with tangible benefits for environmental protection, patient safety, and public health.

## Figures and Tables

**Table 1:** Distribution of collected pharmaceutical dosage forms in 2023, 2024, and 2025 expressed as percentage and total weight (kg).

Pharmaceutical Form	2023	2024	2025
Tablets	63.4% (577.9 kg)	57.8% (385.5 kg)	59.6% (338.8 kg)
Capsules	11.3% (103.0 kg)	18.6% (124.1 kg)	11.4% (64.8 kg)
Powders	3.5% (31.9 kg)	4.1% (27.3 kg)	4.4% (25.0 kg)
Ointments	2.1% (19.1 kg)	1.5% (10.0 kg)	3.5% (19.9 kg)
Solutions/Injectable solutions	8.2% (74.7 kg)	14.7% (98.0 kg)	3.5% (19.9 kg)
Suspensions	4.9% (44.7 kg)	3.4% (22.7 kg)	3.5% (19.9 kg)
Ophthalmic solutions	– (0 kg)	– (0 kg)	2.6% (14.8 kg)
Patches	– (0 kg)	– (0 kg)	4.4% (25.0 kg)
Creams	2.1% (19.1 kg)	4.1% (27.3 kg)	0.9% (5.1 kg)
Syrups	4.5% (41.0 kg)	4.5% (30.0 kg)	0.9% (5.1 kg)
Non-medication products	0.4% (3.6 kg)	0.3% (2.0 kg)	4.4% (25.0 kg)

Percentages correspond to the relative proportion of total annual collected weight for products that met analytical inclusion criteria. Values in kilograms represent total weight collected per year. Non-medication products were collected but not included in dosage-form percentages. A dash (–) indicates no products were recorded for that form in a given year. Total weight collected: 911.5 kg (2023), 667.03 kg (2024), 568.4 kg (2025).

**Table 2:** Regulatory classification of medications (2023–2025).

Category	2023 (kg, %)	2024 (kg, %)	2025 (kg, %)
Prescription medications (Frac. II)	5.47 kg (0.6%)	8.68 kg (1.3%)	14.78 kg (2.6%)
Over-the-counter medications (Frac. IV)	617.00 kg (67.8%)	549.29 kg (82.35%)	388.79 kg (68.4%)
Homeopathic medications (Frac. V)	16.41 kg (1.8%)	4.34 kg (0.65%)	10.23 kg (1.8%)
Herbal medicines/Supplements (Frac. VI)	190.50 kg (20.9%)	104.66 kg (15.69%)	114.82 kg (20.2%)
Unregulated/Non-medication products	72.92 kg (8.0%)	—	39.79 kg (7.0%)

The data correspond to the grouping of regulatory fractions defined by Mexican standards: Fraction II: Prescription medications. Fraction IV: Over-the-counter (OTC) medications. Fraction V: Homeopathic medications. Fraction VI: Herbal medicines or dietary supplements, the “Unregulated / Non-medication products” category includes items without an official health classification. Values are expressed in kilograms (kg) and as the percentage relative to the total collected each year.

**Table 3:** Distribution of collected pharmaceutical waste according to ATC therapeutic group (2023–2025).

ATC Group	2023% (kg)	2024% (kg)	2025% (kg)
M—Musculoskeletal system	38.1% (347 kg)	19.2% (128 kg)	21.0% (119.36 kg)
A—Alimentary tract and metabolism	—	—	18.3% (104.02 kg)
C—Cardiovascular system	0.5% (5 kg)	—	19.3% (109.70 kg)
N—Nervous system	0.1% (1 kg)	—	10.5% (59.68 kg)
J—Systemic anti-infectives	—	—	4.4% (25.01 kg)
L—Antineoplastic and immunomodulating agents	—	—	3.5% (19.89 kg)
D—Dermatologicals	—	—	3.6% (20.46 kg)
H—Hormones	1.1% (10 kg)	—	1.6% (9.09 kg)
P—Antiparasitic products	1.0% (9 kg)	0.3% (2 kg)	2.6% (14.78 kg)
R—Respiratory system	0.1% (1 kg)	—	5.3% (30.13 kg)
S—Ophthalmological/Otological preparations	—	—	0.6% (3.41 kg)
V—Various	1.1% (10 kg)	0.4% (3 kg)	—
Without ATC code	13.6% (124 kg)	55.8% (372 kg)	5.3% (30.13 kg)

Percentages are descriptive; year-to-year differences reflect changes in product composition and classification completeness, not statistical comparisons. Percentages correspond to total weight collected of medications meeting analytical criteria. “Without ATC code” includes products that could not be reliably assigned due to incomplete labeling, deterioration, or insufficient identification. Percentages may not sum to 100% due to minor ATC groups not listed. Total weight collected: 911.5 kg (2023), 667.03 kg (2024), 568.4 kg (2025).

**Table 4:** Percentage per therapeutic category based on kg collected per year (2023–2025).

Category	2023 (%) (kg)	2024 (%) (kg)	2025 (%) (kg)
Antibiotics	30.99% (282.7 kg)	31.43% (209.7 kg)	39.52% (224.5 kg)
Antivirals	8.45% (77.0 kg)	2.86% (19.1 kg)	1.86% (10.6 kg)
Antiparasitics	12.68% (115.5 kg)	14.29% (95.4 kg)	20.09% (114.1 kg)
Antifungals	15.49% (141.2 kg)	11.43% (76.2 kg)	18.53% (105.3 kg)
NSAIDs/Analgesics	32.39% (295.8 kg)	40.00% (266.8 kg)	20.00% (113.7 kg)

Percentages were calculated based on the total weight of pharmaceutical waste collected per year (2023 = 911.5 kg, 2024 = 667.03 kg, and 2025 = 568.4 kg). Values in parentheses represent the corresponding kilograms for each proportion.

## Data Availability

The original contributions presented in this study are included in the article. Further inquiries can be directed to the corresponding author.
